# Geometric Symmetry of Dielectric Antenna Influencing Light Absorption in Quantum-Sized Metal Nanocrystals: A Comparative Study

**DOI:** 10.3389/fchem.2018.00494

**Published:** 2018-10-16

**Authors:** Xinyan Dai, Kowsalya Devi Rasamani, Gretchen Hall, Rafaela Makrypodi, Yugang Sun

**Affiliations:** Department of Chemistry, Temple University, Philadelphia, PA, United States

**Keywords:** light scattering, resonant scattering, random scattering, enhanced optical absorption, dielectric antenna

## Abstract

Silica nanoparticles, optically transparent in the visible spectral region, represent a class of dielectric antenna to tune the propagation and local field distribution of the visible light through surface scattering while the energy loss is minimized. The light scattering on the surface of silica nanoparticles include resonant scattering and random scattering that strongly depend on their geometry: spherical silica nanoparticles with the highest geometrical symmetry favors the light scattering resonances on the nanoparticle surfaces to promote resonant scattering while non-spherical silica nanoparticles mainly support random scattering. Both resonant scattering and random scattering of light on the silica nanoparticles are capable of enhancing the light absorption in quantum-sized metal nanocrystals attached to the surfaces of the silica nanoparticles. The contributions of resonant scattering and random scattering to the enhancement of light absorption have been compared and discussed. The understanding highlights the importance of the geometry of the silica nanoparticle antenna on the design and synthesis of composite materials for efficient light harvesting.

## Introduction

A dielectric antenna consisting of a block of ceramic material of varying shapes interacts with electromagnetic waves while loses much less energy than the metal counterparts, resulting in an efficient modulation of the incident waves (Ashkin and Dziedzic, 1981; Ausman and Schatz, 2008; Matheu et al., 2008; Anderson, 2010; Grandidier et al., 2013; Yin et al., 2013; Kuznetsov et al., 2016; Huang et al., 2018). For instance, a dielectric resonator antenna with an appropriate geometry can allow the incident electromagnetic wave to bounce back and forth against the antenna surface, supporting scattering resonances to form new standing waves near the antenna surface, behaving as resonant scattering. The new surface standing waves can possibly radiate and propagate into space if the antenna surface is leaky. In contrast, an incident electromagnetic wave can also (elastically) scatter away from the antenna surface into space regardless of the geometry of the antenna, behaving as random scattering. Therefore, a dielectric antenna supporting different scattering modes leads to a difference in influencing the absorption spectrum of a material that can absorb the incident electromagnetic energy when it is placed near the antenna. The random scattering usually does not alter the absorption spectrum profile of the energy-absorbing material while the resonant scattering does. A typical example extensively reported in literature is the use of silica particles with sizes of several hundreds of nanometers and larger as a class of dielectric antenna to improve the capability of light absorption in active materials of light-harvesting devices such as solar cells (Matheu et al., 2008; Matsko, 2009; Grandidier et al., 2011; Gupta et al., 2018). Since silica is transparent in the visible spectral region and silica nanoparticles do not absorb visible light, the enhanced light absorption in active materials is attributed to the strong light scattering (both resonant scattering and random scattering) on the surfaces of the silica nanoparticles. When the silica nanoparticles are mixed with the light-absorbing active materials to form a composite in a given volume, the light scattering from the surface of the silica nanoparticles elongates the light traveling path to benefit light absorption in the active materials (Matheu et al., 2008; Kumari and Narayana, 2012; Son et al., 2013; Ullah et al., 2015; Rahman et al., 2016). In addition to the light scattered away from the surface of the silica nanoparticles, the light scattering resonances on the surface of the silica nanoparticles also influence the optical response of active materials attached to the silica nanoparticles (Ausman and Schatz, 2008; Grandidier et al., 2011; Liu et al., 2015). The surface scattering resonances usually create electrical fields much stronger than the incident light near the surface of the silica nanoparticles, significantly enhancing the light absorption of active materials attached to the surface of the silica nanoparticles (Zhang et al., 2016; Codrington et al., 2017; Rasamani et al., 2017, 2018; Dai et al., 2018b). Herein, we study the influence of geometry of silica nanoparticles on their light scattering behavior as well as the corresponding enhanced light absorption in quantum-sized metal nanocrystals attached to the silica nanoparticles. Using the quantum-sized metal nanocrystals as the unique probe, the contributions of resonant scattering and random scattering to the enhanced light absorption are compared. The enhanced light absorption in quantum-sized metal nanocrystals, for example, the nanocrystal catalysts made of platinum group metals (PGMs), is beneficial for more efficiently exciting hot electrons in the quantum-sized metal nanocrystals to drive chemical transformations as discussed elsewhere (Wei et al., 2018).

## Materials and methods

### Synthesis of silica nanospheres (SiO_x_ NSs)

Silica nanospheres (SiO_x_ NSs) were prepared through a sol-gel process relying on a controlled hydrolysis and condensation of tetraethyl orthosilicate (TEOS, 98%, Sigma-Aldrich) (Green et al., 2003). An appropriate amount (1.7 mL) of TEOS was added to a solution containing 29.1 mL of absolute ethanol (Pharmco-Aaper), 3.21 mL of deionized (DI) water, and 1.96 mL of ammonia hydroxide (28–30 wt.% in water, Fisher Scientific). The reaction proceeded for 2 h at a stirring rate of 600 rpm to complete the growth of SiO_x_ NSs. Changing the amount of water and TEOS could tune the size of the synthesized SiO_x_ NSs. The resultant SiO_x_ NSs were collected through two cycles of centrifugation and washing with ethanol, and then dried overnight in an oven set at 60°C.

### Synthesis of truncated silica nanospheres (t-SiO_x_ NSs) and ellipsoidal silica nanoparticles (e-SiO_x_ NPs)

The t-SiO_x_ NSs and e-SiO_x_ NPs were synthesized by following the procedure reported in literature with slight modifications (Rahmani et al., 2017). A desirable amount (50–300 mg) of cetyl trimethylammonium bromide (CTAB, 99.5%, Chem-Impex Int'l Inc.) was added to a mixed solvent containing 50 mL of deionized water and 5 mL of ethanol. The temperature of the solution was maintained at 50°C and stirred at 800 rpm for 30 min. To this solution was added 350 μL of NaOH aqueous solution (3 M, Fisher Scientific) to dissolve CTAB completely. While the temperature and magnetic stirring was maintained, adding 575 μL of TEOS to the CTAB solution initiated the nucleation and growth of silica nanoparticles. Continuous reaction for 2 h completed the synthesis of silica nanoparticles. The geometry of the silica nanoparticles was determined by the concentration of CTAB, i.e., 100 mg of CTAB for t-SiO_x_ NSs and 200 mg of CTAB for e-SiO_x_ NPs. The resulting SiO_x_ nanoparticles were collected through multiple cycles of centrifugation (at 6,000 rpm for 10 min) and washing with ethanol. The collected particles were dried overnight in an oven set at 60°C.

### Synthesis of rodlike silica nanoparticles (r-SiO_x_ NPs)

Rodlike silica nanorods (r-SiO_x_ NPs) with different aspect ratios were synthesized and purified based on the method reported in previous studies (Kuijk et al., 2011; Murphy et al., 2016). In a typical synthesis of r-SiO_x_ NPs with an aspect ratio of 0.8, 30 g of polyvinylpyrrolidone (PVP, molecular weight ~55,000, Sigma-Aldrich) was first dissolved in 30 mL of 1-pentanol (99%, Acros Organics) with assistance of sonication in a 50-mL centrifuge tube. To this PVP solution was sequentially added 0.5 mL of absolute ethanol, 1.2 mL of DI water, and 0.3 mL of 0.18 M aqueous sodium citrate dehydrate (Fisher Scientific). The solution was homogenized via vortex for 30 s. 0.4 mL of ammonium hydroxide was then added to the solution followed by vortex for 30 s. To the solution was finally added 0.3 mL of TEOS. The solution was then vortexed for 1 min and maintained still for 1.5 h, forming r-SiO_x_ NPs with the aspect ratio of 0.8. The length and radius (thus the aspect ratio) of r-SiO_x_ NPs could be tuned by changing the amount of ethanol, water, sodium citrate, ammonium hydroxide, TEOS, and reaction time. The synthesized r-SiO_x_ NPs were collected by three cycles of centrifugation (at 6,000 rpm for 20 min) and washing with ethanol and water. The rods were then re-dispersed in ethanol with assistance of sonication for 2 h. The dispersion was centrifuged at 800 rpm to remove larger rods in the sediment, leaving nearly mono-dispersed r-SiO_x_ NPs in the supernatant.

### Synthesis of quantum-sized Pt and Rh nanocrystals

Colloidal Pt nanocrystals were synthesized through reduction of hexachloroplatinate anions in an aqueous solution at ambient condition. In a typical synthesis, 26 mL of aqueous solution of trisodium citrate (4 mM, Alfa Aesar) was added to 50 mL of aqueous solution of hexachloroplatinic acid hexahydrate (0.4 mM, Acros Organics) while the solution was stirred at 800 rpm. To this solution was added 5 mL of aqueous solution of 4 mM sodium borohydride (98%, Sigma-Aldrich) slowly, triggering the reduction of hexachloroplatinate anions to immediately turn the solution from yellow to brown. The reaction lasted 2 h under a continuous stirring, forming a black dispersion containing Pt nanocrystals.

Colloidal Rh nanocrystals were synthesized through a polyol process involving sequential reactions at two different temperatures (Biacchi and Schaak, 2015). In a typical synthesis, 0.238 g of potassium bromide (KBr, Alfa Aesa), 0.088 g of PVP, 0.024 g of sodium hexachlororhodate (III) dodecahydrate (Na_3_RhCl_6_·12 H_2_O, Alfa Aesa) were mixed with 7 mL of ethylene glycol (EG, Fisher Scientific) in a 20-mL glass vial. Warming up the solution at 40°C for 1 h dissolved the reagent powders completely. The solution was then heated up to 90°C and this temperature was maintained for 15 min to initiate nucleation. The reaction was heated to 150°C and maintained for 1 h, facilitating the growth of colloidal Rh nanocrystals. The resulting dispersion of Rh nanocrystals was mixed with 3 mL of acetone/water (9/1 in V/V), followed by centrifugation at 13,400 rpm for 10 min. The settled powders were then re-dispersed with 3 mL of acetone/water (9/1 in V/V). Repeating the centrifugation/re-dispersion cycles for 5 times removed PVP and ions from the dispersion of the Rh nanocrystals.

### Functionalization of silica (SiO_x_) nanoparticles

The synthesized SiO_x_ nanoparticles were functionalized with (3-amniopropyl) triethoxysilane (APTES, 98%, Acros Organics) to introduce positively charged surfaces. An ethanolic dispersion of 2 mg mL^–1^ of silica nanoparticles was first prepared with assistance of ultrasonication. To 10 mL of the silica nanoparticle dispersion was dropwise added 0.1 mL of APTES while the temperature of the dispersion was maintained at 60°C. It took 30 s to complete the addition of APTES. The dispersion was continuously stirred for 8 h, leading to the conjugation of APTES to the surface of the silica nanoparticles. The functionalized silica nanoparticles were collected through centrifugation and washing with ethanol, followed by drying in an oven set at 60°C. As for r-SiO_x_ NPs, the corresponding powders were calcined at 500°C for 3 h to burn off PVP from the r-SiO_x_ NPs. An appropriate amount of the calcinated r-SiO_x_ NPs were then added to ethanol, forming a dispersion with a silica concentration of 2 mg mL^–1^. To 12 mL of the r-SiO_x_ NP dispersion was added 7 mL of aqueous solution of hydrochloric acid (37 wt.%, Fisher Scientific). The dispersion was constantly stirred overnight at room temperature. The pre-treated r-SiO_x_ NPs were then collected by centrifugation and washing with ethanol for further surface modification with APTES.

The APTES-modified silica nanoparticles exhibited positively charged surfaces, to which metal nanocrystals with negatively charged surfaces could be attached through strong electrostatic attractions (Zhang et al., 2016; Rasamani et al., 2017, 2018). In a process of loading 1 wt.% Pt nanocrystals to the silica NPs, 4.21 mL of the as-synthesized Pt nanocrystal solution was slowly added to 10 mL aqueous dispersion of the silica nanoparticles with a concentration of 2 mg mL^−1^. Constantly stirring the dispersion of two types of nanoparticles at 600 rpm for 15 min resulted in the attachment of the Pt nanocrystals to the surface of the silica nanoparticles, forming composite SiO_x_-NP/Pt particles. The obtained SiO_x_-NP/Pt particles were collected via centrifugation for 10 min at 6,000 rpm, followed by drying in an oven set at 60°C for 2 h. The same method was also used to load the synthesized Rh nanocrystals to the APTES-modified silica nanoparticles. The loading of metal nanocrystals was tuned by controlling the amount of metal nanocrystals and silica nanoparticles used in the synthesis.

## Materials characterization

Transmission electron microscopy (TEM) images of metal nanocrystals were recorded on a microscope (JEOL JEM-1400). Scanning electron microscopy (SEM) images of silica nanoparticles and silica/metal composite particles were characterized with a field-emission microscope (FEI Quanta FEG 450) that was operated at an acceleration voltage of 20 kV. Inductively coupled plasma atomic emission spectroscopy (ICP-OES, Thermo Scientific 7000 Series) was used to determine the loading contents of metal nanocrystals on the silica/metal composite particles. Diffuse reflectance spectroscopy (DRS) was analyzed by an ultraviolet–visible (UV–vis) spectrophotometer (Thermo Scientific, Evolution 220) equipped with an integration sphere.

## Results and discussion

**Figure 1:**
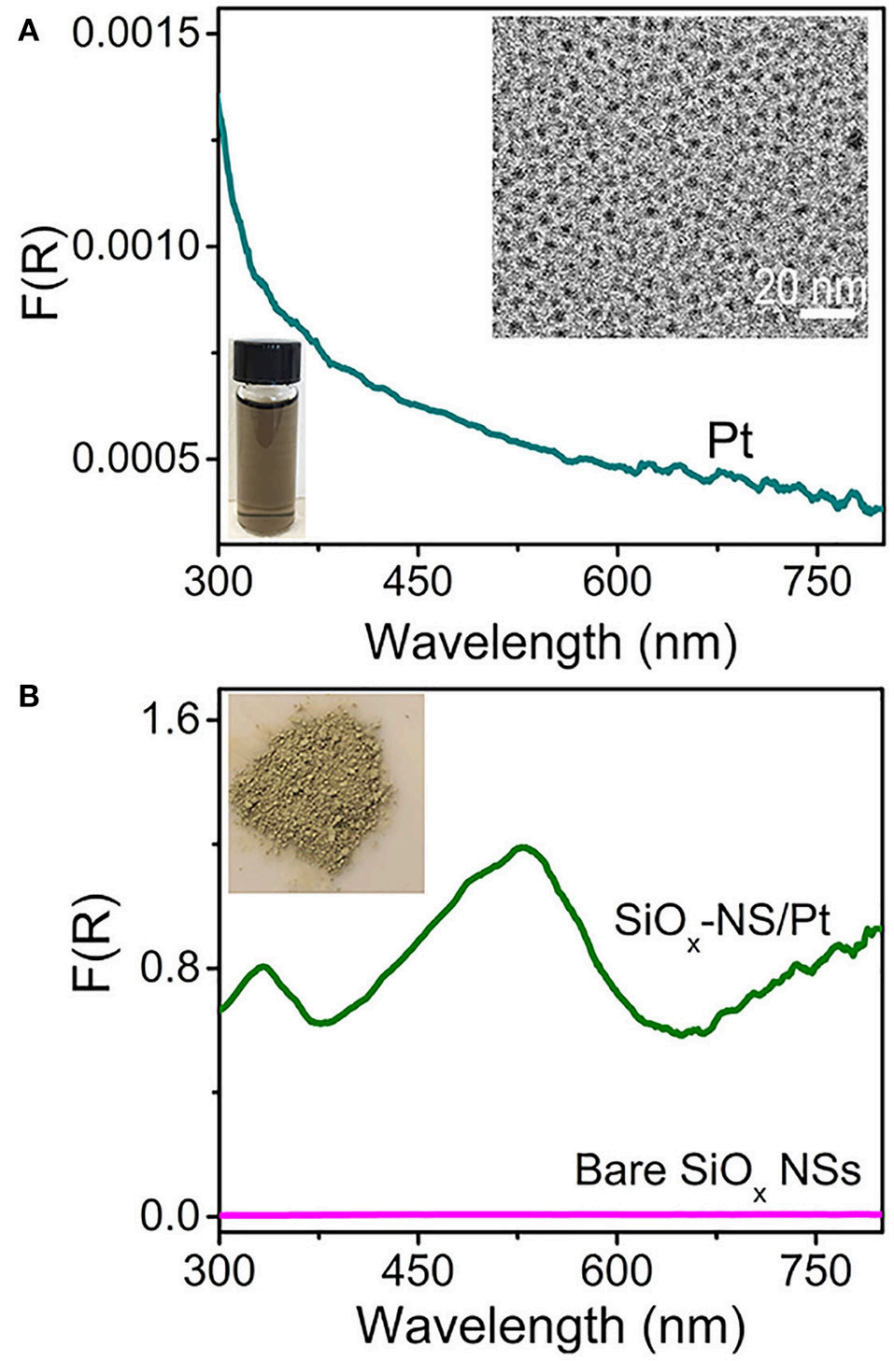
DRS spectra of **(A)** an aqueous dispersion of Pt nanocrystals and **(B)** a powder film of SiO_x_-NS/Pt composite particles. The DRS spectrum of a powder film of SiO_x_ NSs is presented in **(B)** for reference. A representative TEM image of the Pt nanocrystals and a digital photograph of an aqueous dispersion of the Pt nanocrystals are presented as insets in **(A)**. A digital photo of the powders of SiO_x_-NS/Pt composite particles is shown as inset in **(B)**.

Silica nanoparticles with large enough sizes can always produce random scattering in the visible spectral region regardless of their morphology, but generating resonant scattering strongly depends on their morphology. For instance, theoretical modeling and calculations have shown that spherical SiO_2_ particles of several microns supports Fabry-Perot or Whispering Gallery resonances, forming electrical fields near the SiO_2_ surfaces much stronger than that of the incident light (Ausman and Schatz, 2008). Varying the size of the SiO_2_ particles tunes the resonant wavelengths and the enhancement of electrical fields near the particle surfaces. Decreasing the size of silica spheres down to the sub-micrometer scale still supports surface scattering resonances despite the broadness of the resonance peaks. The light scattering resonances on the surfaces of silica nanospheres (SiO_x_ NSs) significantly enhance the optical absorbance and change the spectral profile of absorption in quantum-sized Pt nanocrystals (with size <10 nm) that are attached to the surface of the SiO_x_ NSs (Zhang et al., 2016). An aqueous dispersion of well-dispersed Pt nanocrystals with an average size of 3.2 nm exhibits a brown color and a peakless absorption spectrum in the range of 300–800 nm (Figure 1A). The Pt nanocrystals have negatively charged surfaces, which allows them to attach to the surfaces of positively charged SiO_x_ NSs through the strong electrostatic attraction. This hybridization process results in a uniform distribution of the Pt nanocrystals on the SiO_x_ NSs, forming SiO_x_-NS/Pt composite particles (Figure S1). A powder of the SiO_x_-NS/Pt particles is greenish (inset, Figure 1B), corresponding to the strong light absorption around 530 nm. Figure 1B compares the SiO_x_-NS/Pt particles and the SiO_x_ NSs with regard to the DRS spectra, which exclude the contribution of scattering and are only sensitive to the absorption. The negligible signal of the SiO_x_ NSs indicates that the absorption signal of the SiO_x_-NS/Pt particles originates only from the Pt nanocrystals. Different from the freestanding Pt nanocrystals dispersed in an aqueous solution, the SiO_x_-NS/Pt particles exhibit well-defined peaks, which are consistent with the light scattering resonances on the surface of the SiO_x_ NSs with the highest geometric symmetry (R_3_) (Zhang et al., 2016). The DRS spectrum of the SiO_x_-NS/Pt particles also exhibits a non-zero baseline, more likely independent of the wavelength, which agrees with the feature of random scattering. The comparisons indicate that both resonant scattering and random scattering of the dielectric SiO_x_ NSs are responsible for enhancing the light absorption in the quantum-sized Pt nanocrystals. The respective contribution of resonant scattering and random scattering to the enhanced light absorption is not distinguished.

**Figure 2:**
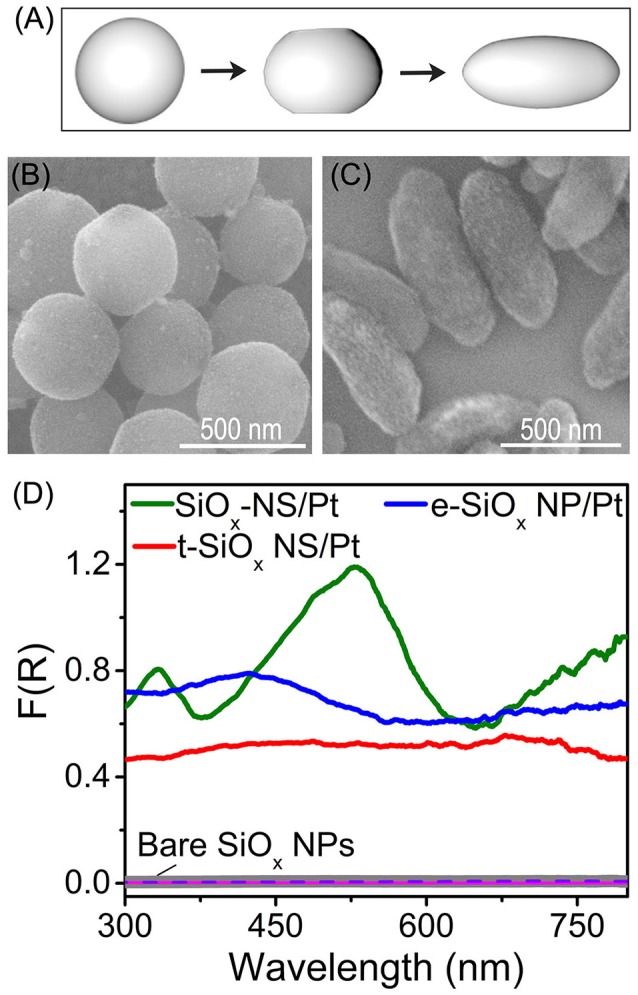
**(A)** Schematic illustration of geometry of SiO_x_ NSs (left), t-SiO_x_ NSs (middle), and e-SiO_x_ NPs (right), highlighting the difference of their geometric symmetries. **(B,C)** SEM images of t-SiO_x_ NSs **(B)** and e-SiO_x_ NPs with Pt nanocrystals loaded on their surfaces. **(D)** DRS spectra of powders of bare silica nanoparticles (SiO_x_ NSs, t-SiO_x_ NSs and e-SiO_x_ NPs) and the corresponding SiO_x_/Pt composite particles with 1 wt.% loading of Pt nanocrystals on the silica nanoparticles.

Theoretical modeling and calculations indicate that the spherical geometry of dielectric particles favors resonant scattering and reducing the geometrical symmetry of the dielectric particles weakens resonant scattering (Gupta et al., 2018). Figure 2A highlights that the geometrical symmetry of silica nanoparticles can be lowered by tuning the concentration of CTAB in the sol-gel reaction as described elsewhere (Figure S2) (Hao et al., 2015; Rahmani et al., 2017). Highly symmetrical SiO_x_ NSs are formed by using the modified Stöber method (Figure S1). Adopting a different synthetic procedure with addition of CTAB yields SiO_x_ with varied morphologies. Addition of a low concentration (4.9 mM) of CTAB to the reaction solution slightly truncates the two poles of SiO_x_ NSs, forming silica nanoparticles (labeled as t-SiO_x_ NSs shown in Figure 2B) with a lower geometric symmetry than the SiO_x_ NSs. Adding more CTAB further truncates the silica nanoparticles and silica nanoparticles with an ellipsoidal shape (labeled as e-SiO_x_ NPs, Figure 2C) are formed with an extremely high concentration (9.8 mM) of CTAB. Same as the SiO_x_ NSs, both the t-SiO_x_ NSs and e-SiO_x_ NPs also exhibit negligible absorption signal in the corresponding DRS spectra (purple and pink curves, Figure 2D). In contrast, the Pt nanocrystals on the t-SiO_x_ NSs and e-SiO_x_ NPs exhibit strong light absorption (red and blue curves, Figure 2D), indicating that both the t-SiO_x_ NSs and e-SiO_x_ NPs are still capable of enhancing light absorption of the Pt nanocrystals due to light scattering of the silica nanoparticles. The corresponding DRS spectra become essentially peakless, implying the absence of resonant light scattering on the t-SiO_x_ NSs and e-SiO_x_ NPs with lowered geometric symmetry of *D*_∞*h*_. The spectral difference of the Pt nanocrystals on the differently shaped silica nanoparticles highlights the importance of geometric symmetry of the silica nanoparticles on determining their light scattering mode. Resonant scattering, which is responsible for the appearance of well-defined intense absorption peaks in the DSR spectra, is very sensitive to the geometric symmetry of silica nanoparticles. Only the SiO_x_ NSs with the highest geometrical symmetry of *R*_3_ supports strong surface scattering resonances. Lowering the geometric symmetry of the silica nanoparticles drastically suppresses the resonant scattering while random scattering is barely influenced (Figure 2D). The difference in the DRS intensity shows that the resonant scattering and random scattering of SiO_x_ NSs make approximately equal contributions to enhance the light absorption in the Pt nanocrystals at the resonance frequencies. At the non-resonance frequencies, the random scattering dominates the enhancement of light absorption in the Pt nanocrystals.

**Figure 3:**
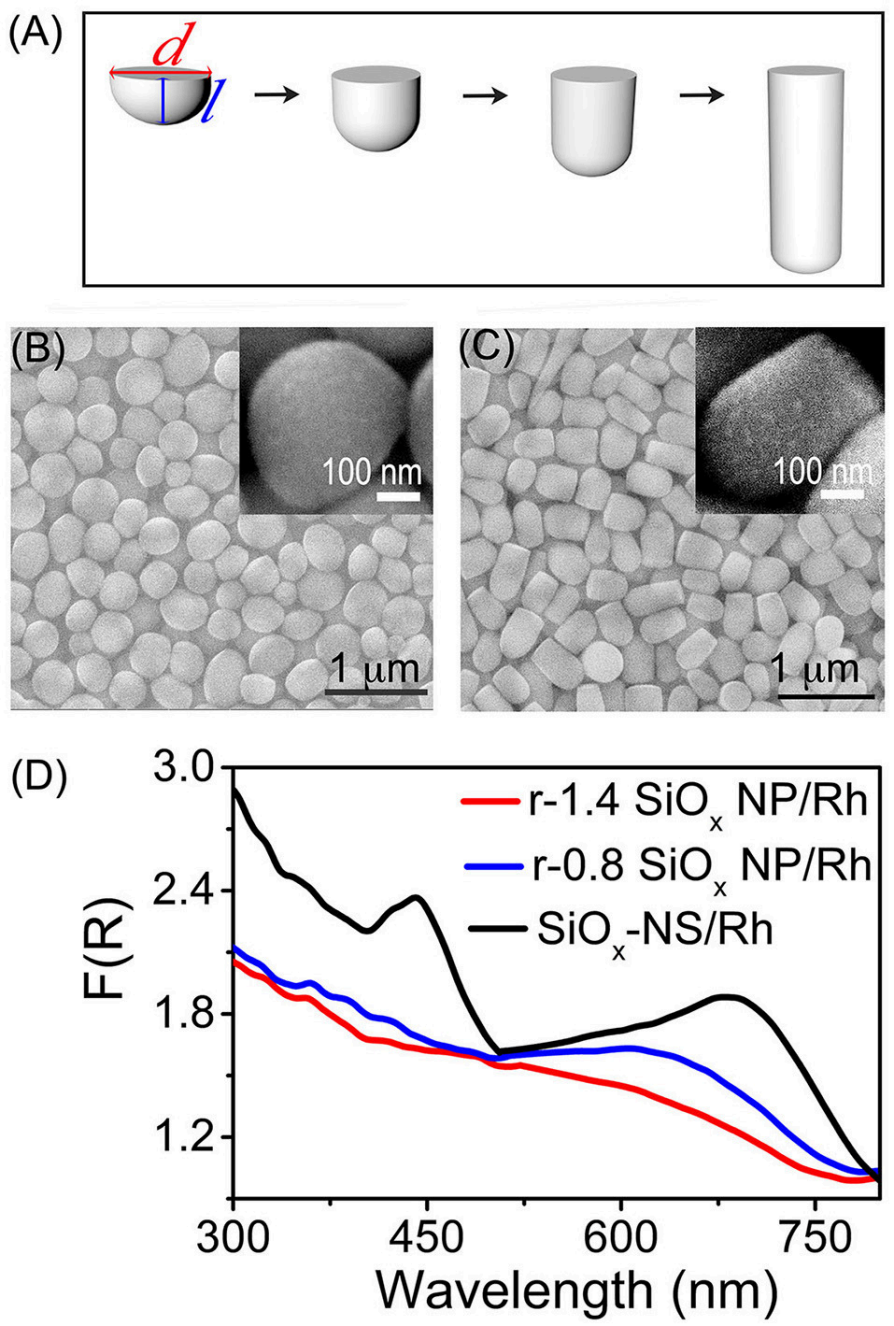
**(A)** Schematic illustration of the geometry of r-SiO_x_ NPs with different aspect ratios. The aspect ratio is defined by the ratio of the length (*l*) to the width (*d*) of an r-SiO_x_ NP. **(B,C)** SEM images of the r-SiO_x_ NPs with aspect ratios of 0.8 (denoted as r-0.8 SiO_x_ NPs) **(B)** and 1.4 (denoted as r-1.4 SiO_x_ NPs) **(C)**. The insets present the SEM images of the r-SiO_x_ NPs covered with Rh nanocrystals. **(D)** DRS spectra of powders of the silica nanoparticles (SiO_x_ NSs, r-0.8 SiO_x_ NPs, and r-1.4 SiO_x_ NPs) with 2 wt.% loading of Rh nanocrystals.

Light scattering on the SiO_x_ NSs can enhance optical absorption in any nanoparticles attached to the SiO_x_ NSs (Zhang et al., 2016; Rasamani et al., 2017, 2018; Dai et al., 2018b). For example, quantum-sized Rh nanocrystals with sizes of ~3 nm exhibit a peakless absorption spectrum while strong light absorption with well-defined peaks is observed from the Rh nanocrystals loaded to the SiO_x_ NSs (with an average diameter of 356 nm, Figure S3). To further verify the importance of spherical symmetry of the silica nanoparticles on resonant scattering, another set of silica nanoparticles with reduced geometrical symmetry are synthesized by controlling the aspect ratio of the rodlike particles. The silica nanoparticles start nucleation at nanosized water-rich emulsion droplets followed by anisotropic growth along the direction away from emulsion droplets, forming rodlike nanoparticles with one flat end and one rounded end (Figure 3A, Figure S4). These rodlike silica nanoparticles (r-SiO_x_ NPs) exhibit a geometric symmetry of *C*_∞*V*_ and their geometric aspect ratio is determined by the lateral dimensions shown in Figure 3A. Similar to the SiO_x_ NSs, these r-SiO_x_ NPs are also feasible to attract the quantum-sized Rh nanocrystals to their surfaces, forming SiO_x_/Rh composites. Figures 3B,C show the SEM images of bare silica and composite samples (insets) formed from the r-SiO_x_ NPs with aspect ratios of 0.8 and 1.4, respectively. The corresponding powders of these composite particles exhibit strong optical absorption although their DSR spectra are different from that of the SiO_x_-NS/Rh composite particles (Figure 3D). The spectral difference shows that the intensity of the absorption peaks decreases as the aspect ratio of the r-SiO_x_ NPs deviates from the unity (i.e., 1) that corresponds to the aspect ratio of the SiO_x_ NSs. This relationship can be quantitatively compared by integrating these DRS spectra in the range of 300–800 nm, and the integrated values are shown in Figure S5 as a function of aspect ratio of the supporting SiO_x_ NPs. The volcano shape with a maximum at the aspect ratio of 1 (corresponding to spherical SiO_x_ NPs) again highlights that the SiO_x_ NSs are more effective in enhancing light absorption in the Rh nanocrystals than the rodlike silica nanoparticles regardless of the aspect ratios >1 or < 1. A more deviation of the aspect ratio weakens the absorption peaks more, further confirming that high geometric symmetry of the SiO_x_ NSs is crucial to support strong resonant scattering on the dielectric silica nanoparticles. Regardless of the aspect ratio of the silica nanoparticles, the featureless baselines of these DRS spectra remain essentially consistent, indicating that the random scattering of the silica nanoparticles makes an approximately constant contribution to enhance the light absorption in the Rh nanocrystals. At the resonance frequencies, the light absorption in the Rh nanocrystals enhanced by the resonant scattering of the SiO_x_ NSs is comparable to the absorption enhanced by the random scattering. The light absorption is dominated by the enhancement originated from the random scattering at the non-resonance frequencies. The DRS spectra shown in Figures 2D, 3D consistently highlight that both resonant scattering and random scattering of the silica nanoparticles are capable of enhancing the light absorption in the quantum-sized metal nanocrystals attached to the silica nanoparticles. The occurrence of resonant scattering strongly depends on the geometric symmetry of the silica nanoparticles. The SiO_x_ NSs with the highest geometric symmetry supports the strongest resonant scattering while the resonant scattering weakens with decrease of their geometric symmetry. In contrast, the random scattering is independent of the geometry of the silica nanoparticles.

**Figure 4:**
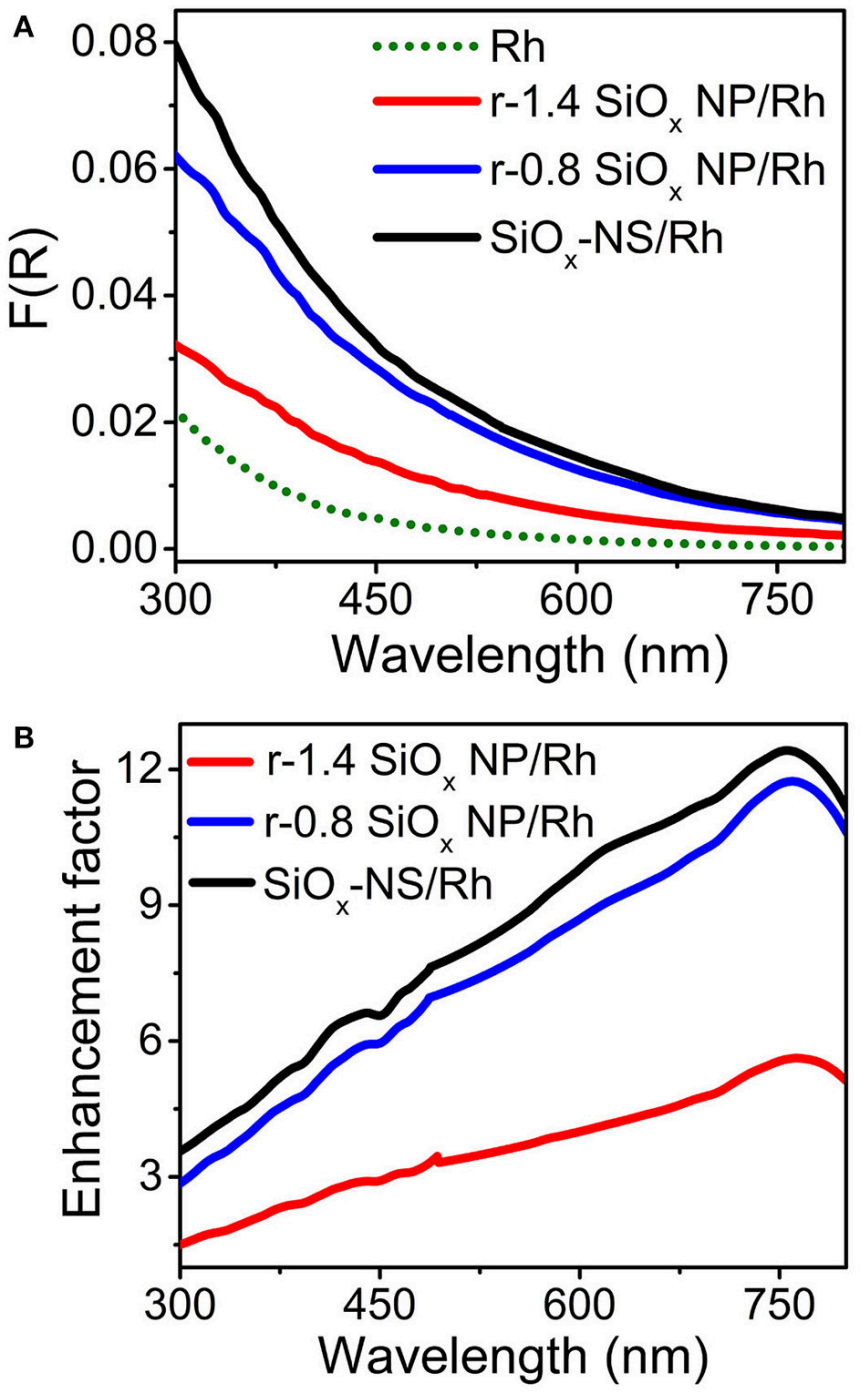
**(A)** DRS spectra of aqueous dispersions of well-dispersed Rh nanocrystals (green dotted) and SiO_x_/Rh composite particles formed from SiO_x_ NSs (black), r-0.8 SiO_x_ NPs (blue), and r-1.4 SiO_x_ NPs (red). The intensity of these DRS spectra were normalized against the mass concentration of Rh nanocrystals. **(B)** Calculated enhancement spectra for the SiO_x_ NSs (black), r-0.8 SiO_x_ NPs (blue), and r-1.4 SiO_x_ NPs (red).

The light scattering efficiency of the dielectric silica nanoparticles also relies on the refractive index (*n*) of the surrounding environment (Mishchenko et al., 2002; Zhu et al., 2010). A large difference of refractive indexes between the surround medium and the silica nanoparticles (*n*_silica_ ≈1.4–1.45 for the sol-gel silica) promotes light scattering. Therefore, the silica nanoparticles dispersed in water (*n*_water_ = 1.33 at room temperature) exhibit a much lower light scattering efficiency compared to the dry powder of the silica nanoparticles in air (*n*_air_ = 1). The variation of refractive index of the surround medium also influences the scattering resonance frequencies of the SiO_x_ NSs. Figure 4A compares the DRS spectra of aqueous dispersions of freestanding Rh nanocrystals and SiO_x_/Rh composite particles formed with varying silica nanoparticles, which exhibit profiles different from the DRS spectra of the corresponding SiO_x_/Rh dry powders (Figure 3D). However, the light absorption in the Rh nanocrystals is always enhanced when the Rh nanocrystals are attached to the silica nanoparticles. The good dispersion of the Rh nanocrystals in water and on the surfaces of the silica nanoparticles ensures that the measured DRS signals represent the accumulation of optical absorption of individual Rh nanocrystals with exclusion of possible interparticular coupling between the adjacent Rh nanocrystals. As a result, the enhancement spectrum, i.e., enhancement factor as a function of wavelength, enabled by the silica nanoparticles can be calculated by dividing the DRS spectrum of a dispersion of the corresponding SiO_x_/Rh composite particles against the DRS spectrum of the dispersion of the freestanding Rh nanocrystals (green dotted curve, Figure 4A). Figure 4B presents the calculated enhancement spectra enabled by the SiO_x_ NSs and the r-SiO_x_ NPs with aspect ratios of 0.8 and 1.4, showing that the maximum enhancement factor can reach ~12 around 750 nm for the SiO_x_ NSs. In the visible spectral region (i.e., at λ > 450 nm), the enhancement factor is always higher than 7 for the SiO_x_ NSs although it is lower for the r-SiO_x_ NPs. Since the light scattering efficiency of the dry SiO_x_ NSs in air is much higher than that of the wet SiO_x_ NSs in solvents (e.g., ethanol, water, etc.), the optical absorption power of the Rh nanocrystals attached to the SiO_x_ NSs could be enhanced by a higher factor when SiO_x_-NS/Rh composite particles are used in gas atmospheres. In order to achieve the maximum scattering-enhanced light absorption in quantum-sized metal nanoparticles, the synthesis of SiO_x_ NSs has to be carefully controlled to ensure the refractive index of the SiO_x_ NSs to reach the possible maximum value (Li et al., 2014; Zulfiqar et al., 2016; Dai et al., 2018a).

## Conclusion

**Figure 5:**
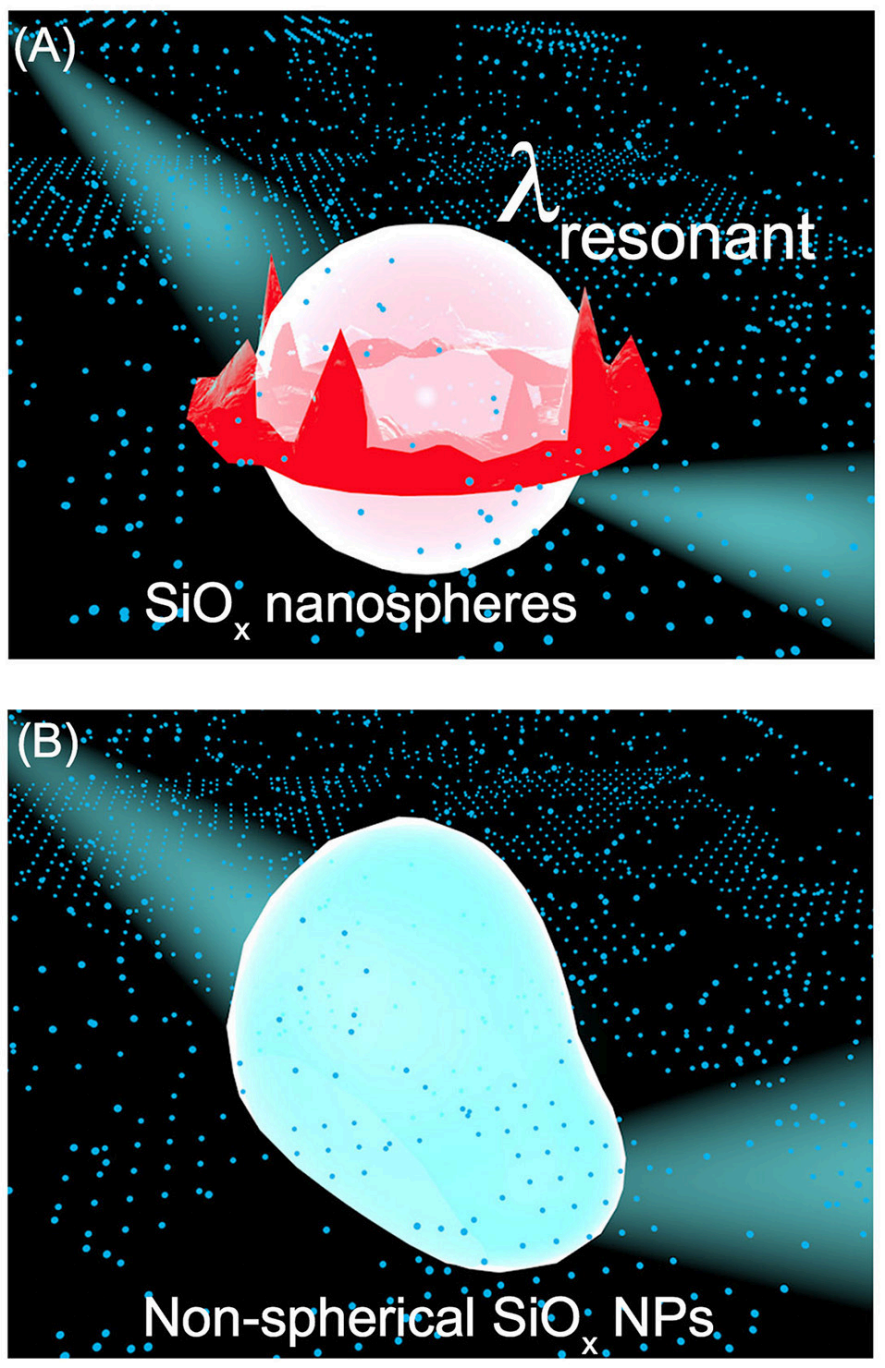
Schematic illustration highlighting the difference of light scattering on silica nanoparticles with different geometric symmetries. **(A)** A SiO_x_ NS with the highest geometric symmetry of R_3_ supports both strong resonant scattering, which generates new standing waves with stronger electric fields near the surface of the SiO_x_ NS, and random scattering, which scatters the incident light off the surface of the SiO_x_ NS in directions different from its original propagation direction. **(B)** A SiO_x_ NP with non-spherical shape, corresponding to a geometric symmetric lower than R_3_, merely supports random scattering. The influence of geometry of SiO_x_ NPs on the light scattering mode can be transferred to the enhanced optical absorption of quantum-sized metal nanocrystals attached to the SiO_x_ NPs as presented in Figures 1–4. The red and blue colors denote high and low electric field intensity, respectively.

In conclusion, silica nanoparticles with lateral dimensions of hundreds of nanometers and larger represent a class of dielectric antenna that does not absorb visible light, exhibiting strong light scattering in the visible spectral region with a minimum energy loss. Regardless of the geometry of the silica nanoparticle antenna, random scattering of the incident light is always observed while the wave function is not altered. When the geometry of the silica nanoparticles exhibits a high enough symmetry, the nanoparticle antenna can also support surface scattering resonances that generate new standing waves with wave functions different from the incident light. For example, light scattering resonances on the surface of SiO_x_ NSs with the highest geometric symmetry result in the formation surface “hot spots,” at which the new electromagnetic waves exhibit higher power density than the incident light. Figure 5 schematically highlights the dependence of scattering mode on the geometry of the silica nanoparticles. The highly symmetric SiO_x_ NSs support both resonant scattering and random scattering while the silica nanoparticles with low geometric symmetry (e.g., r-SiO_x_ NPs with large aspect ratios shown in Figure S4) support merely random scattering. When light-absorbing species are attached to the surface of the silica nanoparticles, their optical absorption power can be enhanced by both random scattering and resonant scattering. The DRS spectra of the SiO_x_/Pt and SiO_x_/Rh composite particles shown in Figures 2D, 3D represent the typical examples highlighting that the optical absorption of quantum-sized metal nanocrystals is significantly enhanced by light scattering (including both random scattering and resonant scattering) on the silica nanoparticles. The random scattering of light from the silica nanoparticles propagates in directions different from that of the incident light, elongating the propagation pathway of light in a material block made of silica/metal composite particles. The longer light pathway allows more incident light to be absorbed by the metal nanocrystals attached on the silica nanoparticles, resulting in an enhancement of overall light absorption. It is worth pointing out that the random scattering does not change the spatial power density of the scattered light. In contrast, resonant scattering of the incident light creates “hot spots” on the surface of SiO_x_ NSs to significantly increase the local electrical fields (corresponding to power density), which enhance the optical (energy) absorption in the metal nanocrystals attached to the surface of the SiO_x_ NSs. The mechanistic difference in enhancing light absorption in quantum-sized metal nanocrystals for random scattering and resonant scattering might be more influential toward non-linear optical properties, for example, photo-excited hot electron generation in the quantum-sized metal nanocrystals (Wei et al., 2018). The understanding sheds light on designing composite materials with the dielectric silica nanoparticle antenna to promote performance of applications.

## Author contributions

YS, XD, and KR: experimental design, data analysis and interpretation, manuscript writing, and manuscript revision; XD, KR, GH, and RM: material synthesis and characterizations, data acquisition.

### Conflict of interest statement

The authors declare that the research was conducted in the absence of any commercial or financial relationships that could be construed as a potential conflict of interest.
