# Supplementary material for: Geometric Symmetry of Dielectric Antenna Influencing Light Absorption in Quantum-Sized Metal Nanocrystals: A Comparative Study
Source: Front Chem. 2018 Oct 16;6:494. doi: 10.3389/fchem.2018.00494 (PMC6198039; doi:10.3389/fchem.2018.00494)
Supplement: Supplementary file 1 [file Data_Sheet_1.pdf]

Supplementary Material

Geometric Symmetry of Dielectric Antenna Influencing Light Absorption in Quantum-Sized Metal Nanocrystals: A Comparative Study

Xinyan Dai[1#], Kowsalya Devi Rasamani[1#], Gretchen Hall[1], Rafaela Makrypodi [1], Yugang Sun[1*]

[1]Department of Chemistry, Temple University, Philadelphia, Pennsylvania, USA

# *These authors contributed equally to this work.*

*** Correspondence:** Dr. Yugang Sun: ygsun@temple.edu

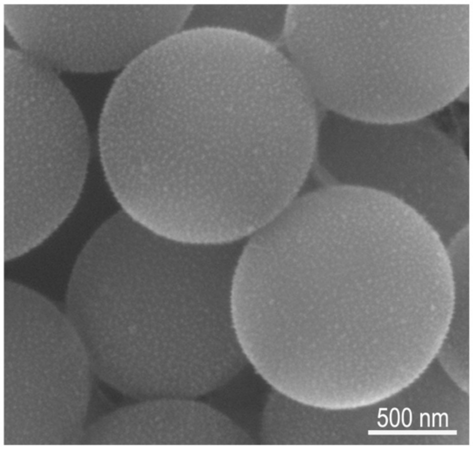

Figure S1 SEM image of SiO_x-NSs/Pt composite nanoparticles with 1 wt.% loading of Pt nanocrystals.

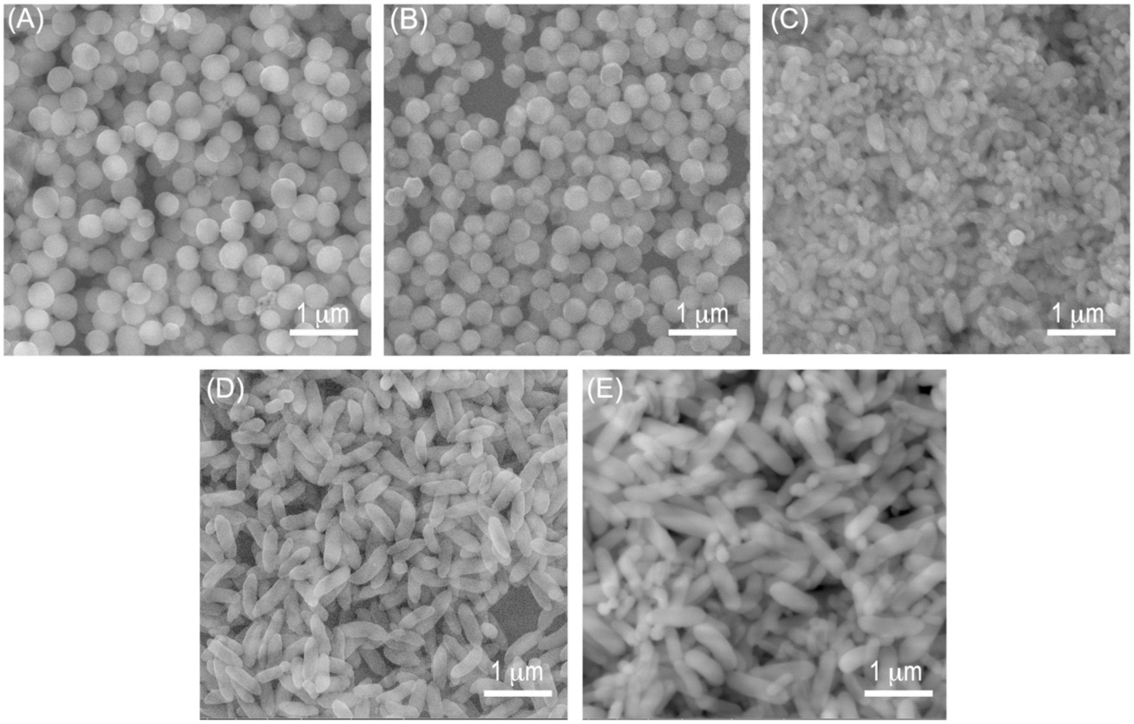

Figure S2 SEM images of SiO_x nanoparticles obtained from the reactions with different amounts of CTAB: (A) 50 mg, (B) 100 mg, (C) 150 mg, (D) 200 mg, and (E) 250 mg. With increase of the concentration of CTAB, the geometry of the SiO_x nanoparticles gradually changes from near sphere (A) to truncated sphere (B) and finally to ellipsoidal nanostructures (C-E).

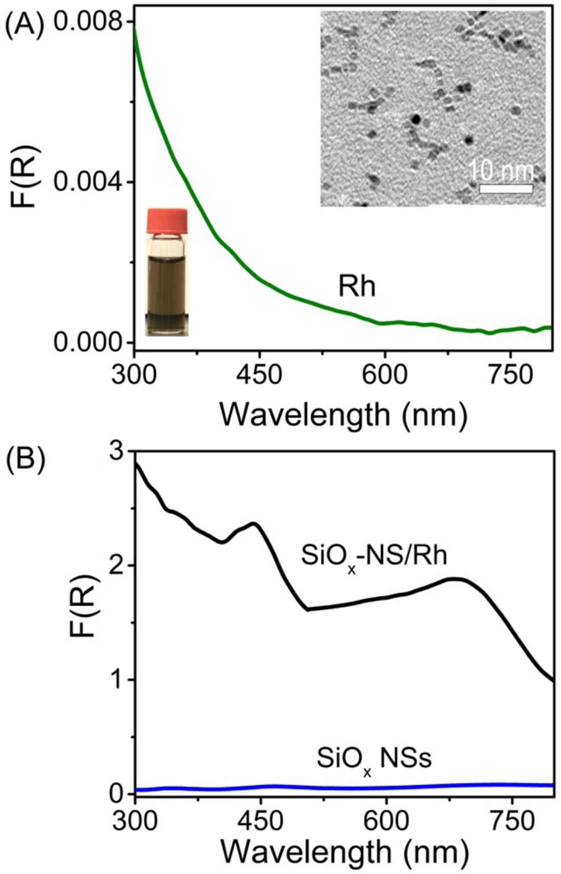

Figure S3 DRS spectra of (A) an aqueous dispersion of Rh nanocrystals and (B) a powder of SiO_x-NS/Rh composite particles with 2 wt.% loading of Rh nanocrystals. A representative TEM image of the Rh nanocrystals and a digital photograph of the aqueous dispersion of the Rh nanocrystals are presented as insets of (A). The DRS spectrum of a powder of the SiO_x NSs is also plotted as reference (blue curve) in (B).

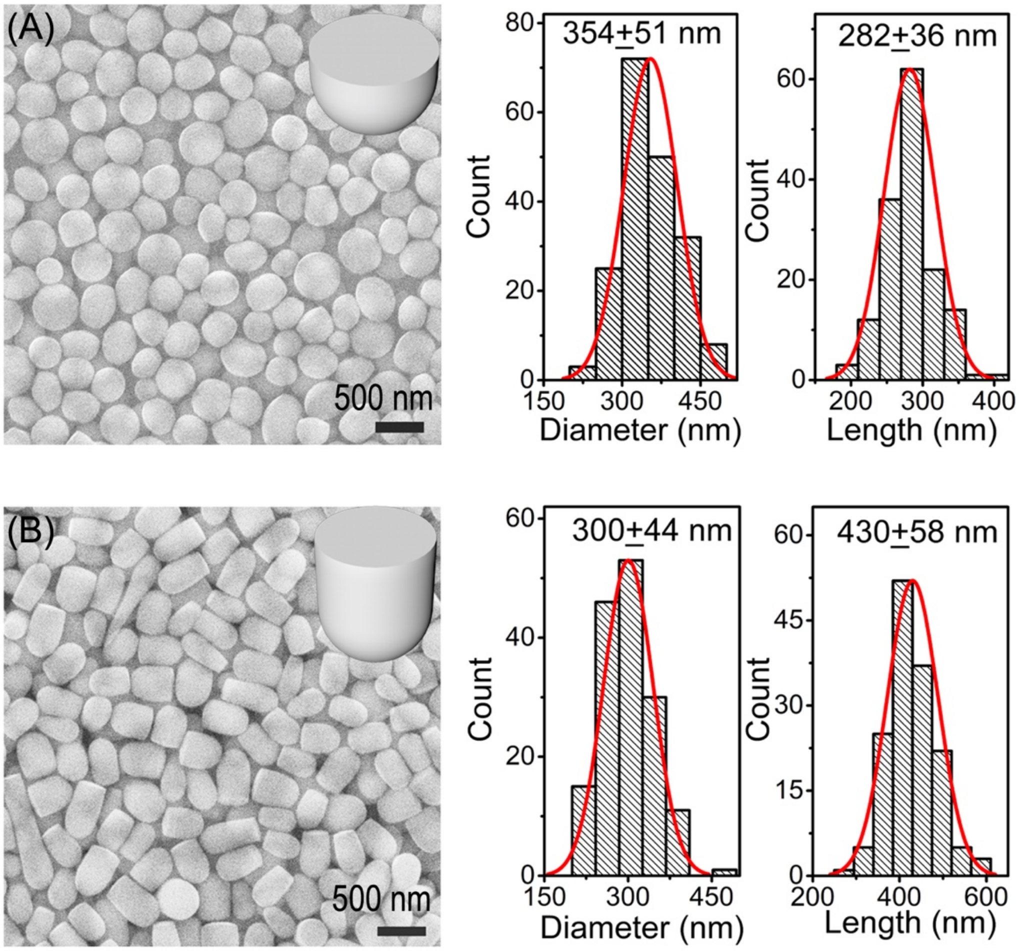

Figure S4 SEM images and statistic histograms of size distributions of rodlike SiO_x nanoparticles with different aspect ratios: (A) 0.8 and (B) 1.4.

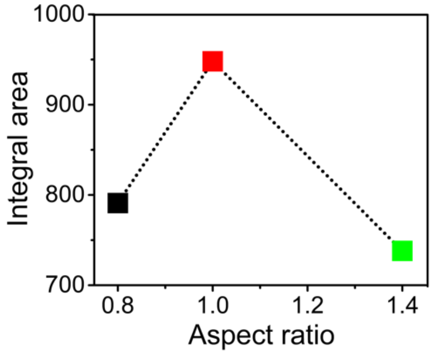

Figure S5 The integrated values of the DRS spectra presented in Figure 3D in the spectral range of 300 nm – 800 nm, showing the dependence of light absorption in the Rh nanocrystals on the geometric aspect ratios of the supporting silica nanoparticles. The volcano-shaped relationship highlights that the spherical silica nanoparticles with an aspect ratio of 1 are more effective in enhancing light absorption in the Rh nanocrystals than the rodlike silica nanoparticles.

frontiers
